# The Top 5 Can’t-Miss Sport Supplements

**DOI:** 10.3390/nu16193247

**Published:** 2024-09-26

**Authors:** Jose Antonio, Flavia Pereira, Jason Curtis, Jose Rojas, Cassandra Evans

**Affiliations:** 1 Exercise and Sport Science, Nova Southeastern University, Davie, FL 33328, USA; 2 Exercise and Sport Science, Keiser University, West Palm Beach, FL 33309, USA

**Keywords:** nutrition, ergogenic aid, performance, exercise

## Abstract

**Background/Objectives:** Sports supplements have become popular among fitness enthusiasts for enhancing the adaptive response to exercise. This review analyzes five of the most effective ergogenic aids: creatine, beta-alanine, nitrates, caffeine, and protein. **Methods:** We conducted a narrative review of the literature with a focus on the sport supplements with the most robust evidence for efficacy and safety. **Results:** Creatine, one of the most studied ergogenic aids, increases phosphocreatine stores in skeletal muscles, improving ATP production during high-intensity exercises like sprinting and weightlifting. Studies show creatine supplementation enhances skeletal muscle mass, strength/power, and muscular endurance. The typical dosage is 3–5 g per day and is safe for long-term use. Beta-alanine, when combined with the amino acid histidine, elevates intramuscular carnosine, which acts as a buffer in skeletal muscles and delays fatigue during high-intensity exercise by neutralizing hydrogen ions. Individuals usually take 2–6 g daily in divided doses to minimize paresthesia. Research shows significant performance improvements in activities lasting 1–4 min. Nitrates, found in beetroot juice, enhance aerobic performance by increasing oxygen delivery to muscles, enhancing endurance, and reducing oxygen cost during exercise. The recommended dosage is approximately 500 milligrams taken 2–3 h before exercise. Caffeine, a central nervous system stimulant, reduces perceived pain while enhancing focus and alertness. Effective doses range from 3 to 6 milligrams per kilogram of body weight, typically consumed an hour before exercise. Protein supplementation supports muscle repair, growth, and recovery, especially after resistance training. The recommended intake for exercise-trained men and women varies depending on their specific goals. **Concluions:** In summary, creatine, beta-alanine, nitrates, caffeine, and protein are the best ergogenic aids, with strong evidence supporting their efficacy and safety.

## 1. Introduction

There is a variety of safe and effective sports supplements. Among them, beta-alanine, creatine, caffeine, nitrates, and protein are some of the most beneficial supplements for trained individuals, offering various performance enhancements. Beta-alanine improves exercise capacity by increasing skeletal muscle carnosine levels, which helps buffer the acid environment in skeletal muscle and thus reduces fatigue. Creatine improves high-intensity performance by replenishing phosphocreatine PCr and ATP stores, improving strength, power, and lean muscle gains. Caffeine enhances alertness and focus and reduces perceived pain during exercise, improving overall performance. Nitrates, present in beetroot juice and leafy greens, boost muscle oxygen delivery, enhancing endurance. Protein is crucial for muscle repair and growth, aiding recovery and promoting skeletal muscle hypertrophy. It is the scientific opinion of the authors of this paper that these five supplements provide the most robust ergogenic effect and have a substantive body of evidence to support their use. Thus, this narrative review aims to provide an update on the scientific evidence vis a vis these five supplements.

## 2. Beta-Alanine

Beta-alanine (BA) is a non-proteogenic amino acid naturally produced in the liver and found in foods like poultry, fish, and meat [1]. It is a precursor to carnosine synthesis, a dipeptide that helps buffer hydrogen ions in skeletal muscles, thereby delaying fatigue during high-intensity exercise [2,3]. Studies have shown that daily supplementation of 4 to 6 g of BA can significantly increase muscle carnosine concentrations. This is beneficial during anaerobic activities, where lactic acid accumulation leads to a drop in muscle Ph [4,5]. Athletes engaged in resistance training and high-intensity exercise tend to have higher muscle carnosine concentrations [4]. However, the response to BA supplementation varies, with some individuals experiencing greater increases in muscle carnosine than others [6]. Factors like baseline carnosine levels, dietary habits, and gender can influence this variability [6,7]. Research has shown that BA supplementation effectively enhances exercise performance in trained and untrained individuals across various demographics, including females and the elderly [7,8]. The primary mechanism of BA’s effectiveness lies in enhancing muscle carnosine concentrations, delaying muscle fatigue by neutralizing hydrogen ions produced during exercise [2]. A typical BA supplementation regimen involves a loading phase of 4 to 6 g daily, divided into smaller doses over several weeks, to maximize carnosine levels [6]. Although paresthesia, a tingling sensation, can occur as a side effect, it is dose-dependent and generally subsides quickly [9]. BA is considered safe and effective for enhancing high-intensity exercise performance, improving training quality, and benefiting a wide range of populations [3,10,11,12,13,14].

### Ergogenic Effects

BA is particularly effective in enhancing anaerobic performance by increasing muscle carnosine levels, which enhances intracellular buffering capacity and delays the onset of muscular fatigue during high-intensity efforts [3]. Research has shown that BA supplementation is most beneficial in activities lasting between 1 and 4 min, where acidosis is a limiting factor, and can significantly improve athletes’ anaerobic power and performance metrics [3,12,15,16]. A study by Shbib et al. [16] investigated the combined effects of plyometric training and BA supplementation on anaerobic power and serum carnosine levels in handball players. The results indicated that average power and peak power increased significantly more in the BA group (10.4% and 9.1%, respectively) compared to the placebo group (−1.7% and −6.8%). Additionally, the serum level of carnosine increased more significantly in the BA group (61.8%) than in the placebo group (43.6%), highlighting BA’s role in enhancing anaerobic performance and muscle carnosine levels. In another study, Sas-Nowosielski et al. [12] explored the effects of BA supplementation on climbing performance. The study revealed that four weeks of BA supplementation significantly improved performance in continuous climbing, lasting about one minute, and repeated bouts of upper body movements. Although the effects were less pronounced in shorter-duration climbing, the BA group showed a significant increase in the total number of moves and time to failure compared to the placebo group, indicating enhanced anaerobic capacity and endurance. Rezende et al. [15] conducted a systematic review and meta-analysis of the muscle carnosine response to BA supplementation. The analysis indicated that BA supplementation substantially increases muscle carnosine content, which plays a crucial role in enhancing anaerobic performance. The study concluded that almost all participants (99.3% response [95% CI]) responded positively to BA supplementation, with significant improvements in muscle carnosine levels and anaerobic performance metrics. Gross et al. [17] (2014) demonstrated that beta-alanine supplementation improved maximal and mean countermovement jump (CMJ) power and aerobic energy contribution in elite alpine skiers, suggesting its effectiveness in sports requiring explosive power. However, the literature evaluating repeated short-duration sprint tasks does not consistently show significant improvements with beta-alanine supplementation. Sweeney et al. [18] reported no significant improvements in power output during repeated five-second sprint bouts. Similarly, Derave et al. [19] found no significant improvements in 400 m sprint times with beta-alanine supplementation, indicating that the benefits of beta-alanine might be limited to longer-duration anaerobic activities. Recently, Guo et al. [20] investigated the effects of combining BA supplementation with short sprint interval training on cardiorespiratory fitness, anaerobic power, and bio-motor skills in volleyball players. Twenty young male athletes were randomly assigned to two equal groups, undergoing 8 weeks of short sprint interval training while taking either 4.8 g of BA daily or a placebo (polydextrose). BA elicited more significant changes in vertical and horizontal jumps than the placebo [20].

Overall, the evidence supports the efficacy of BA supplementation in improving anaerobic performance, particularly in activities lasting over 60 s. By increasing muscle carnosine levels, BA enhances the muscle’s ability to buffer hydrogen ions, reducing fatigue and allowing athletes to sustain high-intensity efforts for longer periods. This makes BA a valuable supplement for athletes engaged in activities that rely heavily on anaerobic glycolysis.

BA supplementation has shown mixed results in improving aerobic performance. It is generally accepted that BA is more effective for high-intensity, short-duration activities lasting 60–240 s than prolonged aerobic exercises [3,21]. Studies have indicated that BA can improve time to exhaustion (TTE) in supramaximal cycling [22], 2 km rowing performance [6,23], and performance in aerobic–anaerobic transition zones in endurance athletes [24], suggesting overall enhanced endurance capacity. For instance, Smith et al. [25] demonstrated significant improvements in TTE among participants who supplemented with BA compared to a placebo group. Ojeda et al. [24] found that both low- and high-dose BA trials (30 mg·kg^−1^ and 45 mg·kg^−1^ of BA) improved physical performance in endurance athletes. Similarly, a study by Baguet et al. [6] reported modest benefits in a 2000 m rowing time trial, showing that while the effect size was small, it could still be meaningful for competitive athletes. However, research on BA’s effects on longer-duration aerobic exercises (over 25 min) is less conclusive. Van Thienen et al. [14] found that eight weeks of BA supplementation (2–4 g per day) substantially improved sprint performance at the conclusion of an intense endurance exercise session. Overall, research indicates that BA offers modest benefits for exercise lasting up to approximately 25 min. Beyond this duration, positive effects are not consistently observed, highlighting the need for more research on long-duration aerobic exercise.

BA supplementation has been extensively studied for its performance-enhancing effects, but its impact on body composition is unclear. The systematic review and meta-analysis by Ashtary et al. [26] indicated that while BA supplementation significantly improves exercise performance, particularly in high-intensity activities lasting 60 to 240 s, its effects on body composition are less pronounced. The meta-analysis found no significant changes in body mass, fat mass, body fat percentage, or fat-free mass with BA supplementation, regardless of the dosage or exercise regimen used. Interestingly, to our knowledge, no studies have examined the effect of beta-alanine combined with diet and exercise on body composition changes as the primary variable. Individual studies have shown mixed results supporting these findings. Kern et al. [27] observed increased lean mass in football players but no significant changes in body composition in wrestlers. Similarly, Glenn et al. [28] reported improvements in lower-body isokinetic strength without changes in body composition in female master athletes. Smith et al. [25] found that BA supplementation combined with high-intensity interval training (HIIT) significantly improved lean body mass in the BA group compared to the placebo group but did not significantly change body fat percentage or mass. This suggests that BA, especially when combined with rigorous training protocols like HIIT, may increase lean body mass but not necessarily reduce fat mass or overall body weight. Overall, the evidence suggests that BA supplementation should be primarily considered for its performance benefits rather than its impact on body composition. While it can enhance certain performance metrics, its effects on altering body composition are less consistent and likely dependent on individual and contextual factors such as the type of exercise regimen and the specific population studied (Table 1).

## 3. Caffeine

Caffeine is the most widely used and well-researched ergogenic aid [30,31,32,33]. Athletes typically consume it to increase performance, mental focus, and perceived energy. Early caffeine research investigated its effects on muscle fatigue. Later research conducted by Costill and colleagues focused on caffeine’s effects on endurance sports. Since then, caffeine’s role in sports performance, cognitive performance, and body composition has increased exponentially. Caffeine naturally occurs in food and beverages such as coffee, chocolate, tea leaves, and kola nuts [31]. It is frequently added to beverages (energy drinks) and can be consumed as a capsule (anhydrous caffeine). In sports, caffeine is most commonly consumed via pre-workouts and energy drinks [30]. Caffeine exerts its effects through multiple mechanisms: the antagonism of adenosine receptors, inhibition of phosphodiesterases, and mobilization of intracellular calcium storage [34]. It acts as an adenosine receptor (A1 and A2A) antagonist [31]. This leads to increased feelings of alertness and concentration. Additionally, caffeine’s inhibitory effects on the adenosine receptors signal the release of dopamine, noradrenaline, and glutamate, leading to positive effects on mood. Caffeine is a nonselective phosphodiesterase (PDE) inhibitor [32]. Inhibition of PDE enzymes in skeletal muscle and adipose tissues increases concentrations of cyclic adenosine monophosphate (cAMP). Elevated cAMP concentrations stimulate hormone-sensitive lipase (HSL) activity and inhibits glycogen phosphorylase activity. This results in increases in fatty acid oxidation instead of glycogen. This glycogen-sparing effect might delay the onset of fatigue. Caffeine stimulates the release and inhibits the reuptake of calcium in the sarcoplasmic reticulum [32]. Caffeine can enhance skeletal muscle contraction by releasing Ca^2+^ ions into the cytosol, aiding skeletal muscle contraction. 

### Ergogenic Effects

Numerous studies have reported positive effects of caffeine in endurance sports, especially running and cycling [30,32,35,36,37]. Dittrich et al. [38] conducted a double-blind, crossover, randomized study examining the effects of caffeinated chewing gum on running performance. After chewing gum, participants completed two exhaustion tests on the treadmill and maximal voluntary contraction of the knee extensor to assess neuromuscular fatigue. Time to exhaustion significantly improved in the caffeine group (40.60 ± 8.53 min) compared to the placebo (33.23 ± 7.41 min). There is no significant difference between the condition and time for neuromuscular fatigue. Interestingly, Hodgson et al. [35] compared caffeinated coffee to caffeine. In this crossover, randomized counter-balanced study, participants (*n* = 8) were randomly assigned to one of four groups: caffeine (5 mg CAF/kg BW), instant coffee (5 mg CAF/kg BW), instant decaffeinated coffee, or placebo. Participants consumed drinks 1 h before completing the exercise protocol, which included 30 min of steady-state cycling followed by a 45 min time trial. No differences in substrate oxidation were reported in any of the groups. Performance times and average power were significantly greater in both caffeine drinks (caffeine: 38.35 ± 1.53 min, coffee: 38.27 ± 1.80 min) compared to decaffeinated (40.23 ± 1.98 min) and placebo (40.31 ± 1.22 min). Guest et al. [36] conducted a study examining the effects of caffeine on endurance exercise and assessed the influence of genotype. Over the course of three visits, participants consumed anhydrous caffeine capsules (2 or 4 mg/kg) or placebo 25 min before warming up for the exercise protocol. The exercise protocol comprised a cycling max exercise test and a cycling time trial. Additionally, participants’ CYP1A2 genotypes were assessed. Cycling time trial times were reduced by 4% in the 4 mg group compared to placebo. No statistical differences were reported between 4 mg and 2 mg and placebo doses. Interestingly, participants with the genotype characterized as a slow metabolizer (CC homozygous, slow) experienced a decrease in performance. In line with previous research, the CYP1A2 genotype predisposes an individual to positive or negative effects of caffeine. These findings confirm caffeine’s positive effect on endurance exercise and suggest that individuals would benefit from personalized caffeine recommendations based on genotype.

Caffeine is beneficial for muscular endurance. However, its effects on muscular strength are less clear. Astorino et al. [39] conducted a study comparing caffeine (6 mg/kg) to placebo on one repetition max (1RM) and repetitions to failure on the bench press and leg press. There were no significant differences between groups in terms of strength outcomes. Tamilio et al. [40] carried out a study on the test–retest reliability and repeatability of caffeine consumption on measures of muscular function. Participants completed six trials (3 with caffeine 3 mg/kg and 3 with placebo) and performed various exercises to measure strength, power, and muscular endurance. Improvements in measures of muscular strength, power, and strength endurance were observed in the caffeine group. However, these findings were inconsistent when comparing individual trials between both groups. Berjisian et al. [41] examined the effects of caffeine intake on resistance exercise and jumping performance. Participants consumed either caffeine (6 mg/g), placebo, or nothing (control) 1 h before completing various exercises. Improvements in muscular strength, endurance, and jump height but not 1RM velocity were reported in the caffeine group. Wu and Jiang [42] explored the effects of consuming different doses of caffeine (CAF) before plyometric jump training (PJT) on sport-related performance and physiological parameters in male basketball players. Twenty-four young athletes were randomly assigned to three groups and completed 6 weeks of PJT while consuming either 3 mg/kg body mass of caffeine (CAF3), 6 mg/kg body mass of caffeine (CAF6), or a placebo (PL) one hour before each training session. Before and after the 6-week training period, the athletes were assessed for basketball-specific performance metrics (vertical jump, 20 m sprint, Illinois change of direction speed [CODS], and maximal strength) and physiological parameters (aerobic capacity and anaerobic power). Comparative analysis of individual responses to training indicated that CAF6 resulted in slightly more significant improvements in vertical jump, maximal strength, and VOmax [42]. Thus, a higher dose of caffeine (6 vs. 3 mg per kg) ostensibly promotes a greater ergogenic effect.

Despite the conflicting studies, meta-analyses and systematic analyses report improvements in strength and muscular endurance following caffeine consumption [43,44] (Table 2). The International Society of Sports Nutrition recommends 3–6 mg/kg of body mass for performance enhancement [30]. For cognitive benefits, doses of 1–3 mg/kg can decrease reaction time, increase speed, and improve memory [45]. Caffeine is quickly absorbed within 1 h of ingestion. It should be ingested 30–60 min before competition/exercise for maximum ergogenic effects. Responses to caffeine vary among individuals due to tolerance, weight, and sensitivity.

## 4. Creatine

Creatine is one of the most widely used ergogenic aids in sports nutrition among athletes [46,47,48]. The most common use of creatine for athletes is to increase intracellular creatine within the muscles, thus increasing creatine phosphate (PCr) availability in the muscles to increase an individual’s capacity for acute exercise in pursuit of increases in training adaptations [49,50,51,52,53,54,55,56]. It has been chiefly used by power or strength athletes for optimal adaptations to training and recovery from PCr exhaustive endeavors [57]. Creatine’s widespread use and extensive research underline its safety and efficacy, making it a popular and effective supplement for athletes and fitness enthusiasts seeking to optimize their training outcomes and overall health.

### Ergogenic Effects

Supplementing creatine has also been reported to aid in recovery from training [58]. Green et al. observed that the ingestion of creatine (5 g) along with 95 g of glucose increased the storage of creatine in skeletal muscles [58]. Creatine retention in the muscles was reported when participants ingested 5 g of creatine along with carbohydrates (47–95 g) and protein (50 g) [59]. Creatine supplementation has been reported to possibly reduce muscle damage and/or amplify recovery from intense bouts of exercise [60]. Other practical benefits from creatine for athletes are a reported reduction in muscle cramping and dehydration, and a possible reduction in musculoskeletal injuries [61]. Athletes using creatine reported less incidence of heat illnesses, muscle cramping, muscle tightness, dehydration, pulled or strained muscles, non-contact-related injuries, and overall injuries, and they had fewer missed days of practice [61]. Yamaguchi et al. demonstrated that 28 days of creatine supplementation (3 g daily) reduced exercise-induced muscle damage [62]. Greenwood et al. similarly reported that football players who used creatine were significantly less likely to experience muscle tightness, muscle strains, muscle cramping, dehydration, heat illness, and total injuries compared to those not supplementing creatine in their diet [63]. In addition, Mills et al. demonstrated that a creatine-supplemented group experienced a more significant increase in leg press, chest press, and total body strength and leg press endurance versus a placebo [64]. Furthermore, creatine supplementation can increase regional muscle thickness [55], enhance muscular endurance in rugby players [53], and increase muscular strength [51,52] and power [50].

In a recent meta-analysis and systematic review, Desai et al. evaluated the additional impact of creatine supplementation on body composition changes during resistance training in adults under 50 years old [65]. A total of 1694 records were screened, and 67 full-text articles were assessed for eligibility, resulting in 12 studies being included in the meta-analysis. Compared to resistance training alone, creatine supplementation increased LBM by 1.14 kg (95% CI 0.69 to 1.59), reduced body fat percentage by −0.88% (95% CI −1.66 to −0.11), and decreased body fat mass by −0.73 kg (95% CI −1.34 to −0.11). No differences were found between training status or carbohydrate subgroups. Training volume was not associated with effect size in any outcomes. Supplementing with 7 g or 0.3 g/kg of body mass of creatine per day is likely to increase LBM by 1 kg and reduce fat mass by 0.7 kg more than resistance training alone [65]. In perhaps one of the most unique investigations on creatine, Aguiar et al. [66] determined whether the belief in having ingested creatine, as opposed to actual creatine ingestion, influenced resistance exercise performance. Fifteen young men (22 years old) completed four exercise sessions, each consisting of three sets of squats and bench presses performed to volitional fatigue at a 10RM load, with 1 min rest intervals between sets. Thirty minutes before each session, participants received one of the following treatments in a randomized order: (1) no supplement (CON); (2) 0.3 g/kg dextrose placebo (PLC); (3) 0.3 g/kg dextrose, labeled as creatine (Cr-False); or (4) 0.3 g/kg creatine, labeled as creatine (Cr-True). Performance was measured by the total number of repetitions completed and the rate of perceived exertion. Interestingly, the investigators concluded that the belief in consuming creatine appears to have a similar impact on acute exercise performance as actual creatine ingestion [66].

For a more extensive review of the literature on creatine, work by Antonio et al. [48], Forbes et al. [47], and Candow et al. [47] is recommended (Table 3).

## 5. Nitrates

Nitrates are bioactive compounds that produce nitric oxide (NO) in the body. Dietary nitrates are found in beetroot juice, pomegranate extract, and green leafy vegetables [67,68,69,70,71] Nitrates were first studied for their role in cardiovascular health and recognized as a signaling molecule [72]. NO’s vasodilation properties led to studying its effects on exercise performance. Due to its purported effects, nitrate supplementation has become popular among endurance athletes. Following consumption, nitrate is first converted to nitrite and then NO. The primary sources of nitrates and nitrites in the body are dietary sources and the L-arginine–NO synthase pathway [73]. NO is also produced from a reduction in nitrate by oral bacteria [74]. Nitrates are vasodilators, increasing oxygen via blood flow to the muscles. This action is thought to improve muscle function, especially during aerobic exercise, when oxygen use is greater [68]. Evidence supports the notion that nitrates influence cellular respiration via an increase in mitochondria biogenesis. Some evidence also supports the notion that nitrates increase the number of glucose transporters and/or insulin availability, resulting in improved adenosine triphosphate (ATP) synthesis [73].

### Ergogenic Effects

Based on the mechanism of action, nitrates are thought to increase exercise efficiency by reducing the energy cost of exercise. Larsen et al. [75] examined the effects of nitrate supplementation on physiological and metabolic parameters during exercise. In this randomized, crossover study, participants were to consume nitrates or a placebo for 3 days, followed by a 10-day washout period. Subjects were instructed to abstain from consuming foods with high–moderate nitrate content. Following supplementation, participants completed submaximal and maximal work tests on a cycle ergometer. Heart rate, lactate, pulmonary ventilation (VE), oxygen uptake (VO_2_), CO_2_ (carbon dioxide) output (VCO_2_), and respiratory exchange ratio (RER) were measured. At submaximal workloads, a reduction in oxygen demand (control: 2.98 ± 0.57, nitrates: 2.82 ± 0.58 L min) was reported. Gross efficiency increased (control: 19.7 ± 1.6%, nitrates: 21.1 ± 1.3%), suggesting the body used energy more efficiently during submaximal work. This study suggests nitrate supplementation improves exercise efficiency during submaximal work.

Baily et al. [76] (2009) conducted a randomized crossover study where participants consumed nitrates (5.5 mmol/day) derived from beetroot juice or a placebo for six days. Moderate and severe exercise tests were performed on days 4, 5, and 6 of supplementation. Following nitrate supplementation, oxygen uptake was reduced (BR: 8.6 ± 0.7 vs. PL: 10.8 ± 1.6 mL·min^−1^·W^−1^) in moderate exercise and severe exercise (BR: 0.57 ± 0.20 vs. PL: 0.74 ± 0.24 L/min). Additionally, participants increased their time to exhaustion (BR: 675 ± 203 vs. PL: 583 ± 145 s). Giv et al. noted that eight weeks of soccer training plus beetroot juice supplementation improved aerobic and anaerobic performance [77].

Yuschen et al. [78] compared the cardiovascular responses to submaximal exercise following either placebo (PLA) or nitrate-rich beetroot juice (NRBRJ) supplementation in healthy men. Twelve healthy men (25 years) participated in 30 min submaximal cycle ergometer exercise trials at 70% of maximal heart rate (HRmax) after consuming either PLA or NRBRJ, in a randomized order. They discovered that acute NRBRJ supplementation combined with exercise proved more effective than PLA supplementation in enhancing aerobic exercise capacity and cardiovascular function in healthy men [78]. Vanhatalo et al. [79] compared the effects of chronic and acute nitrate supplementation on exercise. Participants consumed both placebo and nitrates (5.2 mmol in beetroot juice) for 15 days, with a 10-day washout period between. Blood samples and blood pressure were assessed on days 2, 5, 8, 12, and 15. Participants completed a ramp incremental cycle test and two moderate-intensity step tests 2.5 h after the first dosing and on days 5 and 15. During moderate-intensity exercise, oxygen consumption was lower in the nitrate group at all time points compared to the baseline and on days 5 and 15 compared to placebo. Peak power and work rate at the gas exchange threshold (GET) were significantly higher (*p* < 0.0001) compared to placebo at day 15. Moreover, no significant changes in blood pressure, the oxygen cost of exercise, or exercise performance were observed under placebo conditions, confirming that the benefits were due to the consumption of nitrates via beetroot juice.

The benefits of nitrate supplementation are not limited to aerobic exercise. Mosher et al. [80] examined the effects on resistance exercise. In a double-blind, randomized cross-over design, participants (*n* = 12) consumed beetroot juice (6.4 mmol of nitrates) or placebo with a washout period of 72 h. Based on their 1RM, participants completed three sets of bench press at 60% until failure. A significant improvement in total weight lifted and repetitions to failure were observed. This study demonstrates the positive effects of nitrate supplementation on resistance-training performance.

The studies show that nitrate supplementation is beneficial for sports performance (Table 4). For endurance exercise, nitrates primarily decrease oxygen consumption and improve time to exhaustion. Other studies support the use of nitrates to improve peak power output and resistance training. A limitation of these studies is the small sample size. However, numerous meta-analyses and systematic analyses support these findings [67,68,69,70,71]. Beneficial effects on sports performance are reported in doses ranging from 5 to 16.8 mmol (300–1041 mg) consumed 2–3 h before exercise [81].

## 6. Protein

Protein supplementation has gained significant traction among athletes, bodybuilders, and fitness enthusiasts due to its well-documented benefits in muscle repair, growth, and recovery. This section delves into the mechanisms, dosages, benefits, and potential side effects of protein supplementation, drawing insights from various peer-reviewed studies and authoritative sources. Protein plays a critical role in muscle protein synthesis (MPS), the process by which the body repairs and builds new muscle tissues. Proteins are composed of amino acids, which serve as the building blocks for muscle fibers. Post-exercise, the body enters a state where it requires an adequate supply of amino acids to repair exercise-induced muscle damage and to promote muscle hypertrophy. The process of MPS is highly sensitive to the availability of essential amino acids (EAAs), particularly leucine. Leucine activates the mTOR pathway, a crucial regulator of cell growth and protein synthesis [82]. Studies such as the International Society of Sports Nutrition Position Stand by Jäger et al. [83] emphasize the importance of high-quality protein sources rich in EAAs for maximizing MPS and enhancing muscle recovery and adaptation.

### Ergogenic Effects

Protein supplementation has been shown to significantly enhance muscle mass and strength, particularly when combined with resistance training. Naclerio and Seijo [84] highlight that protein supplementation effectively promotes muscle hypertrophy, especially in individuals engaging in consistent strength training. Protein’s role in providing the necessary substrates for MPS underpins these gains in muscle size and strength. Moreover, post-exercise recovery is crucial for continuous performance improvement and injury prevention. Devries and Phillips [85] discuss how whey protein, a fast-digesting protein source, can expedite recovery by rapidly supplying amino acids to muscle tissues. This quick absorption is beneficial immediately after exercise when the muscles are most receptive to nutrient uptake. In a seminal study by Antonio et al. [86], forty-eight resistance-trained individuals participated in the study, which compared a normal protein (NP) group consuming ~2 g/kg/day and a high protein group (HP) consuming >3 g/kg/day. Both groups followed a split routine, periodized heavy resistance-training program, and tracked their training and diet. The NP group gained more weight, but the HP group had more significant reductions in fat mass and body fat percentage. Both groups showed significant improvements in fat-free mass (FFM), strength, and performance measures, with no significant differences between groups. No adverse changes in blood parameters were observed. This is one of the few investigations that has assessed the effects of a high-protein diet (3.4 g/kg/day) combined with heavy resistance training in trained individuals.

Although typically associated with strength training, protein supplementation benefits other athletes. It helps repair muscle fibers damaged during prolonged activities, thus supporting quicker recovery and reducing muscle soreness. The work of Morgan and Breen [87] indicates that protein hydrolysates can significantly aid in exercise-induced muscle recovery and adaptation. While protein supplementation, such as soy protein, can enhance recovery by reducing exercise-induced muscle stress and inflammation, it does not universally improve performance. For instance, a study on marathon runners found that continuous protein supplementation helped reduce acute stress markers but did not significantly enhance endurance performance [88]. Pre-sleep protein intake among professional cyclists did not provide additional benefits during intense training periods, suggesting that when athletes already consume a high-protein diet, extra supplementation might not yield further performance gains [89].

In a recent investigation, Mhamed et al. [90] randomly assigned 29 well-trained endurance athletes to a whey (30 g/d) or control group. The two groups followed their specific training regimens during the 2-month intervention. Whey protein supplementation combined with two months of endurance training resulted in a reduction in body fat and an increase in leg muscle volume [90]. Kim et al. [91] examined the effects of whey protein supplementation, with controlled diets, on muscle mass and function improvements following resistance training. Thirty-two men were randomly divided into two groups: one receiving whey protein isolate and the other receiving a placebo. Participants were provided meals tailored to their daily energy needs and followed a supervised resistance-training program for 60 min per day, six days per week, over four weeks. After the intervention, the whey protein group showed significantly greater increases in muscle mass and strength improvements in several muscle groups, including the dominant knee and shoulder extensors, compared to the placebo group. The whey protein group also exhibited greater total work output in both the knee and the shoulder extensors [91]. Jacinto et al. [92] discovered that whey protein supplementation was more effective than a leucine-matched collagen peptide supplement in increasing muscle size, but not strength and power, following a 10-week resistance-training program in young adults. In a study on golfers, Seo et al. split golfers into two groups: One group received a mixed protein supplement, while the other received a placebo [93]. The supplement contained casein calcium, whey protein, and isolated pea protein, and participants were instructed to take it once daily for 8 weeks. They found that consuming a mixed protein supplement containing animal and plant proteins positively influenced golf performance and muscle function.

A systematic review comparing soy protein with whey found that soy could provide equivalent benefits in terms of muscle adaptation and antioxidant status but did not consistently outperform whey regarding VO2max or hormonal responses [94]. The optimal dosage of protein varies depending on individual factors such as body weight, training intensity, and overall dietary protein intake. General guidelines suggest that athletes aim for a protein intake of 1.2 to 2.2 g per kilogram of body weight per day to support muscle growth and repair. Distributing protein intake evenly across meals can enhance muscle protein synthesis throughout the day. Consuming 0.4–0.55 g per kilogram of body weight per meal, as suggested by Mazzulla et al. [95], optimizes muscle protein synthesis and prevents periods of amino acid deficiency.

Protein supplementation offers many benefits for athletes and individuals looking to improve their muscle mass, strength, and recovery. By understanding the mechanisms, optimal dosages, and potential side effects, users can make informed decisions to enhance their performance and health. The reviewed literature underscores the efficacy and safety of protein supplementation when used appropriately, making it a valuable tool in the athletic and fitness community [96] (Table 5).

## 7. Summary

Beta-alanine, creatine, caffeine, nitrates, and protein are among the most effective sports supplements that provide various benefits to athletes. Beta-alanine is known for enhancing exercise capacity by increasing carnosine levels, which helps buffer acid in muscles, thereby reducing fatigue. The recommended dose is 2–5 g per day. Creatine enhances high-intensity performance by replenishing PCr and ATP stores and improving strength, power, and lean body mass. The typical dose is 3–5 g per day. Caffeine improves alertness and focus and reduces pain perception during exercise, improving overall performance. The suggested dose ranges from 3 to 6 mg per kg of body weight, taken 30–60 min before exercise. Nitrates in beetroot juice increase oxygen delivery to muscles, thus enhancing endurance. A typical dose of 300–500 mg of nitrate is usually consumed 2–3 h before exercise. Protein is essential for muscle repair and growth, aiding in recovery and promoting skeletal muscle hypertrophy. The recommended intake varies based on activity level; nonetheless, 1.5 to 2.2 g per kilogram of body weight are recommended. Higher intakes may further improve body composition via the loss of fat mass. Incorporating these supplements may help individuals achieve the particular performance and/or body composition goal that they strive for.

## Figures and Tables

**Table 1:** Beta-alanine randomized controlled trials (RCTs).

Reference	Supplement Dose and Duration	Results	Conclusion
Guo W et al., 2024 [20]	4.8 g daily for 8 weeks	Improved vertical and horizontal jumps	β-alanine had a more significant effect on muscular power.
Ojeda Á et al., 2023 [24]	30 mg·kg^−1^ and 45 mg·kg^−1^ for acute trials	Improved performance at maximal aerobic speed in endurance athletes	High doses of β-alanine have a greater effect on performance in aerobic-anaerobic transition zones.
Sas-Nowosielski K et al., 2021 [12]	4 g/day for 4 weeks	Significant improvements in the total number of “slaps” on campus board (intermittent high-velocity climbing), improved performance on easy traverse	β-alanine improved performance in continuous climbing and repeated upper body movements; it was less effective for shorter climbs.
Shbib S et al., 2021 [16]	4 times/day for 4 weeks	Increased average and peak power; greater increase in serum carnosine levels; decreased fatigue index in the placebo group	β-alanine supplementation combined with plyometric training enhances anaerobic power and carnosine levels in handball players.
Jaques M et al., 2019 [23]	3.2 g/day for 4 weeks	Improved body composition and 2 km rowing performance over time; no significant difference between placebo and β-alanine groups	β-alanine supplementation tended to improve rowing performance; further research is needed.
Smith CR et al., 2019 [29]	6.4 g/day for 4 weeks	No significant effects on body composition, muscular strength, muscular endurance, or intermittent sprinting performance	There is little to no impact on body composition parameters or performance in collegiate rugby athletes; more research is needed.
Glenn JM et al., 2016 [28]	800 mg + 8 g dextrose, 4 times/day for 28 days	Significant increase in total work and peak torque in lower-body isokinetic strength; no effect on handgrip strength or body composition	β-alanine supplementation improves lower-body exercise performance in female master athletes; there is no impact on body composition or handgrip strength.
Bellinger PM et al., 2016 [22]	6.4 g/day for 4 weeks	Improved TTE in supramaximal cycling; no effect on 1 km or 10 km TT performance	Effective for improving TTE in supramaximal cycling; limited effects on 1 km and 10 km TT performance
Gross M et al., 2014 [17]	4.8 g/day for 5 weeks	Improved maximal and mean (CMJ) power, reduced oxygen deficit, and enhanced aerobic energy contribution	β-alanine supplementation improved explosive and repeated jump performance in elite alpine skiers.
Kern BD et al., 2011 [27]	4 g/day for 8 weeks	The β-alanine group gained an average 2.1 lb lean mass compared to 1.1 lb for placebo.	β-alanine appears to augment gains in lean mass.
Baguet A et al., 2010 [6]	5 g/day for 7 weeks	Carnosine content increased 45.3% in soleus and 28.2% in gastrocnemius; performance in 2000 m rowing improved by 4.3 s.	β-alanine supplementation improves performance in highly trained rowers.
Derave W et al., 2010 [19]	4.8 g/day for 4 weeks	Carnosine content increased significantly in calf muscles; improved knee extension torque but no effect on 400 m sprint time	β-alanine increases muscle carnosine content and attenuates fatigue in dynamic contractions but does not improve 400 m sprint performance in well-trained track-and-field athletes.
Van Thienen R et al., 2009 [14]	2–4 g/day for 8 weeks	β-alanine increased peak power output by 11.4% and mean power output by 5.0% during final sprints following a simulated cycling race.	β-alanine supplementation improves sprint performance during endurance cycling, particularly during high-intensity efforts after prolonged exercise.

Legend: TT: time trial, TTE: time to exhaustion, CMJ: countermovement jump.

**Table 2:** Caffeine RCTs.

Reference	Supplement Dose and Duration	Results	Conclusion
Wu S and Jiang H 2024 [42]	3 vs. 6 mg per kg one hour before training for 8 weeks	The 6 mg per kg dose worked best.	The higher dose of caffeine improved aerobic power, strength, and vertical jump.
Tamilio RA et al., 2022 [40]	3 mg/kg consumed 45 min before exercise	Increases in countermovement jump (CMJ), drop jumps (DJ), and repetitions to failure	Acute caffeine ingestion is beneficial for muscular strength, power, and muscular endurance, but it may not have the same effect every time.
Berjisian E et al., 2022 [41]	6 mg/kg caffeine ingested 60 min before exercise	Increases in weight lifted during 1RM, repetitions to failure, and CMJNo differences in 1RM velocity	Caffeine supplementation can improve muscular strength, endurance, and jump height.
Dittrich N et al., 2021 [38]	300 mg caffeine ingested immediately before exercise	Increase in time to exhaustion	Caffeine improves time to exhaustion in running.
Guest N et al., 2018 [36]	0, 2, or 4 mg/kg caffeine ingested 25 min before warmup	4 mg/kg decreased cycling time by 4% compared to placebo	Caffeine improves cycling time.
Hodgson AB et al., 2013 [35]	5 mg/kg consumed 1 h before exercise	4.3% improvement in cycling time trial	Caffeine is an effective ergogenic aid in cycling.
Astorino TA et al., 2008 [39]	6 mg/kg consumed 60 min before exercise	No differences between 1RM and repetitions to failure	Caffeine does not have an ergogenic effect on muscular strength or endurance.

**Table 3:** Creatine RCTs.

Reference	Supplement Dose and Duration	Results	Conclusion
Aguiar MS et al., 2024 [66]	Acute consumption of real creatine vs. fake creatine vs. placebo (dextrose) vs. control (nothing)	Results showed that all treatments led to more repetitions than control for both squat and bench press.	The belief in consuming creatine had the same effect as creatine itself.
Yamaguchi S et al., 2024 [62]	3 g of creatine or placebo for 28 days	The creatine group had less muscle soreness.	Creatine has the potential to enhance recovery vis a vis muscle soreness.
Mills S et al., 2020 [64]	Participants received 0.0055 g/kg of creatine or placebo for 6 weeks.	The creatine group improved significantly in chest press, leg press, total body strength, and leg press endurance while the placebo group remained statistically the same.	Creatine had a positive effect on both muscle strength and endurance in participants who were resistance training.
Candow DG et al., 2011 [55]	6-week study with two placebo groups and 1 group consuming 0.15 g/kg of body weight and another group consuming 0.10 g/kg of body weight	Muscle thickness around the elbow grew significantly in both creatine groups compared to placebo groups. Men on creatine saw greater increases in leg press in comparison to women on creatine.	Creatine during resistance training shows benefits in muscle growth around the elbow compared to placebo.
Chilibeck PD et al., 2007 [53]	8-week supplementation of creatine 5 g or placebo	Increased repetitions for both bench press and leg press in the creatine group	Creatine helps increase muscle endurance in rugby players.
Hoffman J et al., 2006 [52]	10-week study on the effects of creatine 5 g, creatine 5 g with beta-alanine, or placebo	Lean mass and body fat percentage were improved in the creatine plus beta-alanine group, greater strength improvements were seen in both the beta-alanine with creatine groups, and increased testosterone was seen in the creatine-only group.	Creatine and beta-alanine in combination seem to have the greatest effects on lean body mass and fat percentage.
Bemben MG et al., 2001 [51]	9-week study involving a placebo group, a control group, and another group that supplemented with 20 g a day of creatine for five days, and then with 5 g daily for the remainder of the study	Increased cell hydration in the creatine group versus the placebo and control groups.	Creatine may enhance cell hydration.
Stone MH et al., 1999 [50]	5 g creatine for one group, or 60% calcium pyruvate and 40% creatine for the other creatine group in comparison to a calcium pyruvate group only, and a placebo group for a 5-week period	The creatine and the creatine with calcium pyruvate groups showed significantly greater increases for body mass, lean body mass, one repetition maximum (RM) bench press, combined 1RM squat and bench press, and static vertical jump (SVJ).	Creatine alone and creatine mixed with calcium pyruvate increased training adaptations associated with body mass/composition, maximum strength, and SVJ.

**Table 4:** Nitrate RCTs.

Reference.	Supplement Dose and Duration	Results	Conclusion
Yuschen X et al., 2024 [78]	Acute consumption of beetroot juice pre-submax cycling for 30 min	Beetroot juice improved mean exercise load.	Beetroot improved aerobic exercise capacity and cardiovascular function.
Giv V et al., 2024 [77]	8 weeks of soccer training plus beetroot juice supplementation	The beetroot juice supplement significantly changed aerobic power, respiratory exchange ratio, anaerobic threshold, anaerobic power, field performance, and fatigue index.	Soccer players may benefit from supplementing with beetroot juice.
Mosher SL et al., 2016 [80]	6.4 mmol/day for 6 days in the form of beetroot juice	Increase in reps to failure and total weight lifted	Nitrates improve resistance-training performance.
Vanhatalo A et al., 2010 [79]	5.2 mmol/day for 15 days in the form of beetroot juice	Decreased oxygen consumption and blood pressure Increased peak power output and work rate at the gas exchange threshold	Both acute and chronic nitrate supplementation lowers blood pressure, and improves oxygen efficiency and exercise performance.
Bailey SJ et al., 2009 [76]	5.5 mmol/day for 6 days in the form of beetroot juice	Decrease in oxygen consumption Increase in time to exhaustion	Nitrate supplementation lowers oxygen cost and improves tolerance to exercise.
Larsen FJ et al., 2007 [75]	3 days of 0.1 mmol/kg/day nitrates or placebo	Decrease in O_2_ demandNo differences in HR, lactate, V_E_, V_E_/VO_2_ or RER at submaximal workloads	Nitrate supplementation decreased oxygen demands in submaximal work.

**Table 5:** Protein RCTs.

Reference	Supplement Dose and Duration	Results	Conclusion
Mhamed MB et al., 2024 [90]	30 g of whey daily for 2 months	Whey > control for body fat reduction and leg muscle volume.	Whey protein supplementation combined with 2-month endurance training leads to a reduction in body fat and increased leg muscle volume. ASAT, ALAT, and CK were also reduced.
Seo J-W et al., 2024 [93]	8 weeks of a combined animal and plant-based protein (60 g/d)	Golfers improved driver distance, driver ball speed, and grip strength.	The intake of a mixed protein containing both animal and plant proteins positively affected golf performance and muscle function.
Kim CB et al., 2023 [91]	4 weeks of supplementing with whey vs placebo	The whey protein group showed significantly greater increases in muscle mass and strength improvements in several muscle groups, including the dominant knee and shoulder extensors.	Whey protein supplementation enhances muscle mass, strength, and endurance.
Jacinto JL et al., 2022 [92]	10 weeks, whey protein supplementation vs. leu-matched collagen peptides	Whey protein had a greater increase in biceps brachii and vastus lateralis m. thickness	Whey protein supplementation was more effective than leucine-matched collagen peptide supplement in increasing muscle size, but not strength and power, following a 10-week resistance training program in young adults.
Naclerio F & Seijo M 2022 [84]	Varies, typically 20–40 g post-exercise	Significant muscle hypertrophy and strength gains	Protein supplementation, especially post exercise, supports muscle hypertrophy and strength in individuals engaged in regular strength training.
Morgan & Breen 2021 [87]	Varies protein hydrolysates used	Significant aid in exercise-induced muscle recovery and adaptation	Protein hydrolysates are beneficial for quick recovery and adaptation post exercise due to their rapid absorption and effective amino acid delivery.
Mazzulla M et al., 2020 [95]	20–40 g post-exercise, distributed intake (0.4–0.55 g/kg/meal)	Optimized muscle protein synthesis throughout the day	Even distribution of protein intake across meals enhances muscle protein synthesis and prevents amino acid deficiencies.
Antonio J et al., 2015 [86]	3.4 vs. 2.3 g/kg/d	No difference in LBM gain; higher dose resulted in greater fat mass loss	High protein intakes promote fat mass loss.
Devries MC & Phillips CM 2015 [85]	20–40 g post-exercise, fast-digesting protein (whey)	Faster recovery, improved muscle repair and growth	Whey protein is highly effective for post-exercise recovery due to its rapid absorption and high leucine content.
